# A POLICY ANALYSIS OF ELDER ABUSE PREVENTION LEGISLATION: TRANSLATION OF NATIONAL INITIATIVES TO THE STATE LEVEL

**DOI:** 10.1093/geroni/igac059.1597

**Published:** 2022-12-20

**Authors:** Autumn Decker, Kathryn Bruzios, Raven Weaver, Cory Bolkan

**Affiliations:** Washington State University, Pullman, Washington, United States; Washington State University, Pullman, Washington, United States; Washington State University, Pullman, Washington, United States; Washington State University Vancouver, Vancouver, Washington, United States

## Abstract

Elder abuse (EA) is an under-recognized global public health threat in need of enhanced research and policy attention, with 1 in 6 older adults experiencing at least one form of abuse/neglect. Public policy changes, such as the federal 2017 Elder Abuse Prevention and Prosecution Act (EAPPA), aim to address EA, but little is known regarding translation and implementation of the initiatives to state-levels. Applying a thematic analytic approach, we identified four major themes from the EAPPA: criminal investigation support; abuse reporting; financial exploitation; and training/technical assistance. Then, we conducted a comparative policy analysis to evaluate each state’s EA statutes enacted since 2017 to determine whether statutes were updated to reflect the EAPPA’s goals. Preliminary findings from 10 states (i.e., states with the greatest proportion of older adults in each Health & Human Services region) showed that eight states had updated statutes relevant to an identified theme(s). Eight states updated statutes related to financial exploitation, highlighting the growing concern over this specific type of abuse. Four states updated statutes related to reporting, reflecting problems with identifying and detecting elder abuse. Two states updated statutes regarding investigation, demonstrating minimal emphasis on elder abuse investigation. No states updated statutes relevant to training/technical assistance. These findings indicate significant state-level variability in translation and implementation of recent legislation. More recognition, research, and policy attention to EA, in the U.S. and globally, is needed. We conclude with recommendations for innovation and prevention policy efforts coupled with multidisciplinary collaborations to strengthen implementation against EA at the state-level.

